# Psychological distress among Ukrainian displaced persons 2 years after the full-scale invasion: a contextual benchmark comparison

**DOI:** 10.3389/fpubh.2026.1858781

**Published:** 2026-08-19

**Authors:** Ekta Sidhar, Jenny Phillimore, Madeleine Ingham, Olga Andrushchakevych, Heather D. Flowe

**Affiliations:** 1 School of Psychology, University of Birmingham, Edgbaston, Birmingham, United Kingdom; 2 Social Policy, University of Birmingham, Edgbaston, Birmingham, United Kingdom

**Keywords:** anxiety, depression, psychological distress, trauma, Ukrainian refugees

## Abstract

**Introduction:**

This study characterized depression, anxiety, and stress symptoms in a convenience sample of Ukrainian displaced persons approximately 2 years after the full-scale invasion and explored associations with selected settlement-related indicators.

**Methods:**

A cross-sectional online survey was conducted between February and August 2024 among displaced Ukrainian adults (*N* = 241 with complete DASS-21 data), who completed the Depression Anxiety and Stress Scales-21 in Ukrainian, Russian, or English. Mean subscale scores were contextualized using published general-population reference values from the United Kingdom, Portugal, Poland, and Italy, and exploratory associations with housing stability, employment status, and host-country language proficiency were examined.

**Results:**

Mean depression, anxiety, and stress scores fell within the mild to moderate range and were generally higher than available European reference values, although these benchmark comparisons should be interpreted cautiously because the reference samples differed in timing, sampling frame, country context, and gender composition. A supplementary sex-composition sensitivity analysis using Polish female reference values suggested that the depression comparison was robust, whereas anxiety was attenuated and stress was modest. Within-sample analyses showed no meaningful association between distress and either language proficiency or employment status. Housing stability showed a tentative association with anxiety, driven primarily by lower anxiety among participants reporting long-term rather than medium-term housing.

**Discussion:**

These findings suggest continuing psychological distress among Ukrainian displaced persons in this convenience sample, while underscoring the need for longitudinal, representative research that directly measures trauma exposure, uncertainty, and structural settlement conditions.

## 1 Introduction

Conflict and associated displacements create profound psychological consequences, precipitating post-traumatic stress disorder (PTSD), depression, anxiety, and general psychological distress in affected populations (1). Direct exposure to violence and secondary exposure through media coverage or concern for others can elevate psychological distress and mental health symptoms in conflict-affected populations among adults and children (2, 3), while displacement is associated with loss, grief, chronic stress, and systemic uncertainty (4).

Psychological distress in displaced populations is commonly assessed using standardized symptom measures [e.g., Depression Anxiety and Stress Scales-21 (DASS-21, 5)] in diverse populations and language contexts (5, 6). Civilians in war zones often experience equal or greater psychological distress than military personnel. As many as 90% of contemporary war victims are civilians caught in violence (7, 8). A recent meta-analysis found aggregated prevalences of 29% for depression, 31% for anxiety, and 23% for PT*SD* in conflict-affected populations (8). In that review, civilian populations showed higher rates of depression and anxiety than military populations. Other studies of conflict-affected civilians, including internally displaced persons and refugee children, similarly document substantial psychological distress following war exposure and displacement (3, 9–11). Refugees and internally displaced persons represent an especially high-risk group since they are often caught up in conflict, face treacherous journeys to safety and are parted from everything they know and love (12). Systematic reviews indicate that at least 20% of long-settled refugees continue to meet criteria for mental health disorders years after resettlement (13) with post-migration conditions having a powerful effect on mental health (14). Research with Syrian refugees in a Canadian study found that 24% of their cohort screened positive for PT*SD*, with 12 and 27% scoring in severe ranges for depression and anxiety, respectively (15). These elevated rates persist due to ongoing stressors including unemployment and social isolation (1, 2, 16). However, there is also evidence that positive integration processes and outcomes can moderate the effects of prior trauma (17), but these effects have been identified where refugees have been resettled and have permanent leave to remain or a legal pathway to permanence.

Russia’s February 2022 full-scale invasion of Ukraine triggered Europe’s largest displacement crisis since World War II, with over 5 million refugees from Ukraine fleeing abroad by 19 April (18) with about 4 million registered for temporary protection or similar national protection schemes in Europe by mid-September 2022 (19). The speed of displacement was unprecedented, with one million people fleeing in just the first week (20). UNHCR’s data (18) also showed that there were an estimated 7.7 million internally displaced and another 8 million in urgent need of humanitarian assistance and protection in April 2022.

Early empirical data following the invasion of Ukraine reveals severe psychological impacts: a spring 2022 cross-sectional study found Ukrainian respondents reported significantly higher DASS-21 scores than other countries, with nearly half exceeding cutoffs for high depression (46.5%) and anxiety (46.3%) (1). Early studies conducted shortly after the invasion also documented elevated distress among refugees and internally displaced Ukrainians (21). More recently, large-scale population-based research has confirmed a substantial mental health burden both among Ukrainians remaining in the country and among those displaced abroad (22). Another large survey of over 8,300 displaced Ukrainians found approximately 69% met screening criteria for generalized anxiety disorder, with those remaining in Ukraine showing even higher symptom levels than refugees abroad (23). The situation of Ukrainians in Europe is unique in recent times in that they have been hosted in unprecedented numbers and been given large scale support to integrate into their host countries, but unlike resettled refugees they have no pathway to permanence (24).

However, much of the existing evidence was collected either during the early stages of displacement or focuses primarily on estimating prevalence rather than examining the influence of post-displacement settlement factors that may shape longer-term psychological outcomes. Research with asylum-seekers has shown that longer waiting times for decisions about legal status are associated with poorer self-reported emotional and physical health (25). Although displaced Ukrainians in Europe are in a different legal position from asylum-seekers, this literature highlights the potential relevance of continuing displacement and uncertainty for mental health. The role of uncertainty in the psychological health of forced migrants from Ukraine remains underexplored.

The present study explores psychological distress in a convenience sample of Ukrainian displaced persons approximately 2 years after displacement. Post-migration conditions are consistently identified as important social determinants of refugee mental health, particularly where they affect security, autonomy, social participation, and access to resources (14). In addition, we explore whether commonly cited post-displacement settlement indicators (housing stability, employment and host-country language proficiency) are associated with psychological distress within this sample.

Accordingly, this study addresses two questions: (1) how do depression, anxiety, and stress scores in this sample of Ukrainian displaced persons compare with published European general-population norms; and (2) within this sample, are settlement-related indicators (housing stability, employment and host-country language proficiency) associated with levels of psychological distress?

## 2 Method

### 2.1 Study design

This study employed a cross-sectional online survey design to examine psychological distress among forcibly displaced Ukrainians. Depression, anxiety, and stress were assessed using the DASS-21, and selected demographic and settlement-related variables were collected to describe the sample and support exploratory analyses. Ethical approval was granted by the Science, Technology, Engineering, and Mathematics Committee at the University of Birmingham.

### 2.2 Sampling and participants

The survey was designed and hosted on Qualtrics and was available in English, Ukrainian, and Russian. Participants were able to select their preferred language at the beginning of the survey. Study information and a link to the survey were posted on various online platforms (designated study webpage hosted by the University of Birmingham, Facebook, Twitter and LinkedIn) and circulated by the authors within their institutions, networks and among organizations supporting displaced Ukrainian migrants. Recruitment was further facilitated by snowball sampling, whereby individuals who had completed or were aware of the study were encouraged to share it within their networks. All posts were available in English, Ukrainian, and Russian. Participants chose to take part through voluntary self-selection in line with the eligibility criteria, which required participants to be at least 18 years old and to identify as having been forcibly displaced from Ukraine. The survey did not collect a precise date of displacement. Therefore, the timing of data collection was approximately 2 years after the February 2022 full-scale invasion, but we do not have an individually measured duration of displacement for each participant.

Given that participants were accessing the survey from various countries, a link to The United Nations Human Rights Council (UNHRC) support page, which details available support in 99 countries, was provided in the study debrief and on the designated study webpage. Due to the anonymous nature of the study, no compensation was provided for participating. The survey was open between February and August 2024.

Of the 529 participants initially recruited, 280 (52.93%) did not complete the DASS-21. Participants were excluded if they failed to complete all items of the DASS-21, as missing data on this primary outcome measure would compromise the validity of comparative analyses with normative samples. Data were screened for response quality prior to analysis. Participants showing clear indicators of invalid responding, including invariant response patterns across symptom measures and nonsensical item responses, were excluded following manual review; eight participants were excluded following this screening process. Our 54.4% attrition rate is comparable to the 42.8% dropout reported by Xu et al. (26) among Ukrainians surveyed during the March 2022 invasion period and is in keeping with attrition documented in conflict-affected populations where dropout rates range from 40 to 70% (27, 28).

**Figure 1:**
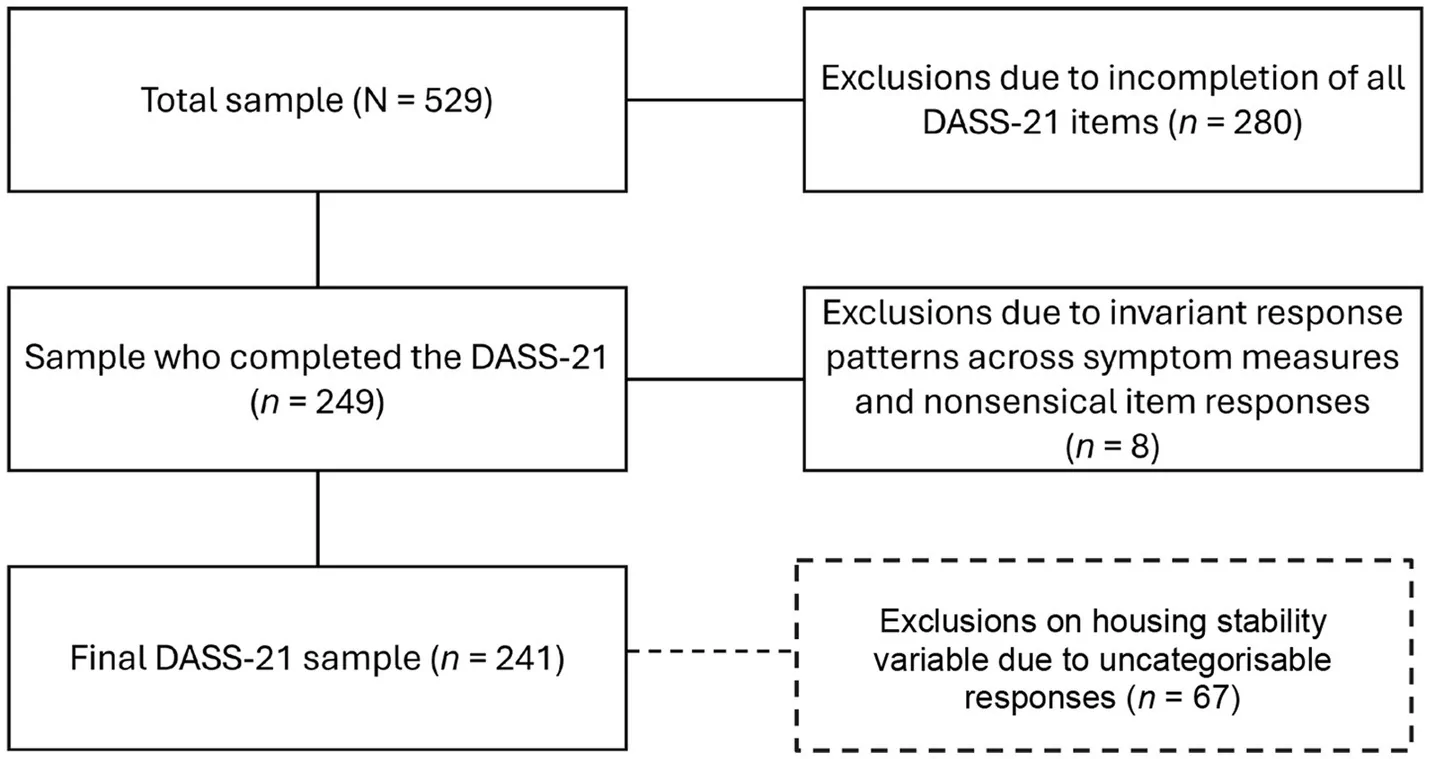
Flow diagram illustrating participant exclusions and final DASS-21 analytic sample.

This resulted in a final sample of 241 participants, ranging in age from 18 to 73 years (*M* = 39.52, *SD* = 10.46) (see Figure 1). The gender distribution was 80.50% female (*n* = 194), 18.67% male (*n* = 45), 0.41% non-binary (*n* = 1), and 0.41% preferred not to say (*n* = 1). The sample gender composition is in alignment with the proportion of Ukrainians in Europe who are women (72.3%) (29). See Table 1 for the distribution of participants across host countries and within Ukraine.

**Table 1:** Distribution of participants (*n* = 241) by country of residence at time of survey.

Host countries	Number of participants
United Kingdom	73
Germany	36
Spain	26
Poland	25
Portugal	15
Croatia	9
Slovenia	7
Internal displacement within Ukraine	6
Other	44

Countries listed in order of frequency. Low frequency countries compiled into ‘Other’ category for brevity.

## 3 Materials and procedures

### 3.1 Measures

#### 3.1.1 Depression, anxiety, and stress scale - 21 (DASS-21)

The DASS-21 (30) is a 21-item self-report that measures the severity of an individual’s psychological distress and symptoms of depression, anxiety, and stress. It is scored on a 4-point Likert scale, with each item assigned a numerical score ranging from 0 (“did not apply to me at all”) to 3 (“applied to me very much or most of the time”). Subscale scores are summed and multiplied by two to align with the full DASS scoring system.

Following standard DASS interpretive guidelines, multiplied subscale scores were interpreted using the following severity bands: depression normal 0–9, mild 10–13, moderate 14–20, severe 21–27, and extremely severe 28+; anxiety normal 0–7, mild 8–9, moderate 10–14, severe 15–19, and extremely severe 20+; and stress normal 0–14, mild 15–18, moderate 19–25, severe 26–33, and extremely severe 34+. These categories were used descriptively and should not be interpreted as diagnostic classifications.

In this study, the total score of the DASS-21 demonstrated excellent internal consistency [*α* = 0.948; (31)]. The internal reliability for the subscales was as follows: depression (*α* = 0.891), anxiety (*α* = 0.867), and stress (*α* = 0.913).

A confirmatory factor analysis was conducted as a psychometric check of the expected three-factor structure of the DASS-21 in this multilingual sample. The standard correlated three-factor model demonstrated good fit to the data, χ^2^(186) = 367.4, *p* < 0.001, CFI = 0.974, TLI = 0.970, RMSEA = 0.064, SRMR = 0.055, with strong factor loadings across items. This analysis was not a test of measurement invariance across language versions or between the present sample and the published benchmark samples. Accordingly, benchmark mean differences should be interpreted cautiously, as they may partly reflect differences in language, cultural context, or response patterns as well as differences in psychological distress.

The survey also collected demographic and settlement-related information, including age, gender, country of residence, educational attainment, employment status, self-rated host-country language proficiency, and housing stability. Housing stability was assessed using a free-text item asking how long participants could remain in their current accommodation. Ukrainian and Russian materials were prepared with input from Ukrainian-speaking collaborators and checked for clarity before survey launch.

### 3.2 Procedure

First, participants viewed an information sheet available in English, Russian and Ukrainian, outlining the study details and gave informed consent via Qualtrics. Upon consent, participants were asked to create a self-generated alphanumeric ID number. Participants were asked to retain this ID number should they wish to withdraw their data. Participants were able to withdraw their data via an anonymous Qualtrics withdrawal form within 30 days of completing the survey. This ensured that withdrawals could be processed anonymously, without collecting names or other identifiable information. No face-to-face interaction took place during the study, and only non-identifiable demographic details (e.g., age and gender) were collected. Participants completed the survey and were presented with a debrief page upon completion.

### 3.3 Data analyses

All analyses were conducted in R 4.3.2 (32). Descriptive statistics (*n*, *M*, *SD*, minimum, and maximum) for each settlement indicator and DASS-21 subscale were obtained with the functions *summarize*() from the *dplyr* package [v 1.1.4; (33)], *describe*() from the *psych* package [v 2.3.9; (34)], and *readxl* for data import. Confirmatory factor analysis was conducted using the lavaan package [v0.6–17; (35)], with reliability estimates obtained using the semTools package. Participants with incomplete DASS-21 data were excluded from analyses involving DASS-21 outcomes. For demographic and settlement variables, analyses used available cases; therefore, sample sizes vary across analyses depending on item-level exclusions due to uncategorizable responses (see Figure 1). The dataset analyzed for this study can be found in the Open Science Framework at https://osf.io/2qr95/.

#### 3.3.1 Benchmark comparisons

Published DASS-21 reference values from the United Kingdom (36), Portugal (37), Poland (38), and Italy (39) were used to contextualize depression, anxiety, and stress scores in the Ukrainian displaced sample. In the absence of pre-war Ukrainian DASS 21 reference data and comparable benchmark data from Ukrainian displaced populations or closely matched refugee samples, published European general population reference values were used to contextualize the observed symptom levels. These benchmark comparisons were intended to situate symptom levels rather than to estimate population prevalence or infer effects of displacement.

Because raw data from the reference studies were unavailable, comparisons were conducted using published summary statistics: means, standard deviations, and sample sizes. Two-sample Welch t-tests were used because they do not assume equal variances across samples. Twelve benchmark comparisons were conducted in total, reflecting three DASS-21 subscales across four reference countries. A Bonferroni-adjusted threshold of *α* = 0.00417 (i.e., 3 subscales × 4 reference countries = 12 comparisons; 0.05/12 = 0.00417) was therefore used for these comparisons.

Accordingly, *p*-values from these benchmark comparisons are reported as descriptive indicators of the size and consistency of differences relative to available reference values, rather than as definitive inferential tests of displacement-related differences. This is because the reference studies differed from the present sample in sampling frame, country context, year of data collection, gender composition, and language of administration.

Because the Ukrainian sample was predominantly female, we also examined whether sex-specific reference values were available in the benchmark studies. Sex-specific DASS means and standard deviations were available only for the Polish reference sample. We therefore conducted a supplementary sex-composition sensitivity analysis comparing the pooled Ukrainian sample with Polish female reference values. This analysis was intended to assess whether the benchmark pattern was likely to be driven solely by the high proportion of women in the Ukrainian sample, while avoiding unstable sex-stratified estimates within the Ukrainian sample. This sensitivity analysis is reported in Supplementary Material S2.

#### 3.3.2 Settlement variable analysis

Settlement-related indicators were operationalized using three variables: housing stability, local language proficiency and employment status. Housing stability was assessed with the open-ended question, “How long can you remain in this housing?” Responses were translated when necessary and categorized for analysis as uncertain (conditional or unknown durations), short-term (less than 6 months), medium-term (6 months to 1 year), or long-term (more than 1 year). Responses stating ‘indefinitely’ were not categorized, as the meaning of this response could not be consistently mapped onto the predefined temporal groupings. These categories were used as pragmatic indicators of perceived accommodation security, distinguishing participants who reported immediate or short-term arrangements from those reporting longer or more open-ended housing stability.

Local language proficiency was measured through a self-rated score on a 10-point scale, in which participants indicated their perceived current competence in the host country’s language. These variables have been identified as core indicators of integration for refugees (40). Employment status was assessed via a categorical self-report item and grouped for analysis into full-time, part-time, self-employed, unemployed/job-seeking, retired, disabled, other, and not applicable. Unemployed and job-seeking categories were combined due to low cell counts. Kruskal-Wallis tests were used to compare DASS-21 subscale scores across the four housing stability categories. Pearson’s correlation coefficients were used to explore associations between these local language competency and DASS-21 subscale scores. Welch’s ANOVA was used to examine differences in DASS-21 subscale scores across employment groups, with Kruskal-Wallis tests conducted as a non-parametric sensitivity analysis. These approaches were selected to account for unequal group sizes and non-normal distributions.

## 4 Results

### 4.1 Descriptive statistics

Descriptive statistics for the DASS-21 subscales and local language proficiency are presented in Table 2. See Tables 3–5 for housing stability, educational attainment, and employment status descriptives, respectively. Mean scores on the Depression (*M* = 15.55, *SD* = 10.52), Anxiety (*M* = 10.59, *SD* = 9.56), and Stress (*M* = 17.93, *SD* = 10.85) subscales of the DASS-21 correspond to the mild to moderate range of the instrument’s interpretive guidelines (30). Among participants who completed the DASS-21, the largest proportion reported uncertainty regarding their housing stability (38.5%). Within this same subgroup, most participants reported higher educational attainment, including specialist degrees (29.5%) and master’s degrees (32%). Most participants were also in full-time employment at the time of survey completion (36.5%).

**Table 2:** Descriptives statistics for DASS subscales and local language proficiency.

Variable	*n*	*M*	SD
Depression (0–42)	241	15.55	10.52
Anxiety (0–42)	241	10.59	9.56
Stress (0–42)	241	17.93	10.85
Host-country language proficiency	241	5.02	2.54

Scale ranges are shown in parentheses. DASS-21 subscale scores were multiplied by two prior to calculating the overall means and standard deviations for depression, anxiety, and stress, in accordance with recommended scoring procedures that allow comparison with DASS-42 interpretive guidelines.

**Table 3:** Descriptive statistics for housing stability categories.

Category	*n*	*%*
Uncertain	67	38.5
Short-term	14	8.0
Medium-term	56	32.2
Long-term	37	21.3

Percentages are based on participants in the final DASS-21 analytic sample with categorizable housing stability responses (*N* = 174).

**Table 4:** Frequencies of self-reported educational attainment categories.

Category	*n*	*%*
High school	17	7.1
Trade school	14	5.8
Specialist degree	71	29.5
Bachelor’s degree	31	12.9
Master’s degree	77	32
Candidate of Sciences, Ph.D., equivalent or higher	27	11.2
Other	4	1.7

*N* = 241. “Other” includes industrial college/technical school and institute responses. Percentages may not sum to exactly 100 because of rounding.

**Table 5:** Frequencies of self-reported employment categories.

Variable	*n*	*%*
Unemployed/job-seeking	66	27.4
Part-time employed	44	18.3
Full-time employed	88	36.5
Self-employed	18	7.5
Retired	7	2.9
Disabled	1	0.3
Other	17	7.1

*N* = 241.

Self-rated proficiency in the host language fell near the midpoint of the 0–10 scale (*M* = 5.02, *SD* = 2.54). Sample sizes differ across variables because of sporadic item non-response; complete case *n*’s are reported in Tables 1–5.

### 4.2 Participant completion and missingness

Among participants with available data, DASS-21 completion was not significantly associated with age, self-rated host-country language competency, education, housing stability, or gender. The employment comparison showed weak evidence of association but was sensitive to sparse cells and should not be overinterpreted. Country data were missing for 223 participants, all of whom were DASS-21 non-completers. Among participants who provided country information, country group was not significantly associated with DASS-21 completion, *χ*^2^(8) = 4.89, *p* = 0.769. Full analyses are reported in Supplementary Material S1.

### 4.3 Benchmark comparisons

Benchmark comparisons were conducted using the completed DASS-21 analytic sample (*N* = 241). Mean DASS-21 scores in the Ukrainian displaced sample were higher than the corresponding published European reference means across all three subscales, although the magnitude of the difference varied by reference country and subscale.

For depression, the Ukrainian displaced sample mean (*M* = 15.55, SD = 10.52) was higher than the reference means from the United Kingdom (*M* = 5.66, SD = 7.74), Portugal (*M* = 6.84, SD = 6.97), Italy (*M* = 7.00, SD = 6.40), and Poland (*M* = 9.22, SD = 8.46). Mean differences ranged from 6.33 to 9.89 points. Welch tests indicated that all four depression comparisons were statistically significant: United Kingdom, *t*(275.96) = 14.09, *p* < 0.001; Portugal, *t*(291.60) = 12.23, *p* < 0.001; Italy, *t*(344.50) = 11.45, *p* < 0.001; and Poland, *t*(317.08) = 8.70, *p* < 0.001.

For anxiety, the Ukrainian displaced sample mean (*M* = 10.59, SD = 9.56) was higher than the reference means from the United Kingdom (*M* = 3.76, SD = 5.90), Portugal (*M* = 6.27, SD = 6.43), Italy (*M* = 4.80, SD = 5.20), and Poland (*M* = 8.78, SD = 7.48). Mean differences ranged from 1.81 to 6.83 points. Welch tests indicated significant differences for the United Kingdom, *t*(265.12) = 10.82, *p* < 0.001; Portugal, *t*(293.31) = 6.67, *p* < 0.001; Italy, *t*(323.72) = 8.69, *p* < 0.001; and Poland, *t*(312.91) = 2.75, *p* = 0.006. However, the anxiety comparison with Poland did not survive the Bonferroni-adjusted threshold of *α* = 0.00417.

For stress, the Ukrainian displaced sample mean (*M* = 17.93, SD = 10.85) was higher than the reference means from the United Kingdom (*M* = 9.46, SD = 8.40), Portugal (*M* = 11.77, SD = 7.17), Italy (*M* = 12.80, SD = 7.60), and Poland (*M* = 15.08, SD = 9.07). Mean differences ranged from 2.85 to 8.47 points. Welch tests indicated that all four stress comparisons were statistically significant: United Kingdom, *t*(279.97) = 11.65, *p* < 0.001; Portugal, *t*(291.36) = 8.39, *p* < 0.001; Italy, *t*(377.89) = 6.47, *p* < 0.001; and Poland, *t*(323.64) = 3.77, *p* < 0.001.

After Bonferroni correction, all benchmark comparisons remained statistically significant except the anxiety comparison with Poland. A supplementary sex-composition sensitivity analysis using Polish female reference values found that the depression difference remained clear, whereas the anxiety difference was attenuated and the stress difference was modest. Full details are reported in Supplementary Material S2.

### 4.4 Exploratory associations with settlement indicators

#### 4.4.1 Local language proficiency

Pearson correlations indicated that local language proficiency was not associated with psychological distress in this sample. Correlations between language proficiency and DASS-21 depression, anxiety, and stress were small and non-significant: depression, *r*(239) = −0.02, *p* = 0.729; anxiety, *r*(239) = 0.00, *p* = 0.955; stress, *r*(239) = 0.03, *p* = 0.686.

#### 4.4.2 Employment

Differences in DASS-21 scores across employment groups were examined in the final DASS-21 analytic sample. Employment categories with fewer than 10 participants were excluded from hypothesis testing to improve the stability of estimates. Specifically, eight retired participants and two disabled participants were excluded, resulting in an analytic sample of *N* = 231. There were no statistically significant differences between groups for depression (*p* = 0.184), anxiety (*p* = 0.748), or stress (*p* = 0.652). A non-parametric sensitivity analysis using Kruskal–Wallis tests indicated no significant differences for depression (*p* = 0.292), anxiety (*p* = 0.472), or stress (*p* = 0.633). Overall, employment status was not associated with depression, anxiety, or stress in this sample.

#### 4.4.3 Housing stability

Differences in DASS-21 scores across housing stability categories were examined using Kruskal-Wallis tests. This analysis was conducted on the subset of participants in the final analytic DASS-21 sample who had categorizable housing-stability responses, resulting in an analytic sample of *N* = 174. Depression scores differed significantly across groups, *H*(3) = 9.11, *p* = 0.028 (*ε*^2^ = 0.036), as did anxiety scores, *H*(3) = 12.46, *p* = 0.006 (*ε*^2^ = 0.056). The omnibus test for stress was not statistically significant, *H*(3) = 7.75, *p* = 0.051 (*ε*^2^ = 0.028). Dunn’s *post hoc* tests with Holm correction indicated that anxiety scores differed significantly between the long-term and medium-term housing groups (pHolm = 0.006), with lower anxiety in the long-term group (mean rank = 63.51) than in the medium-term group (mean rank = 98.95). For depression, post-hoc comparisons did not remain significant after Holm correction (smallest pHolm = 0.051 for long-term vs. medium-term; long-term mean rank = 65.66, medium-term mean rank = 93.81).

## 5 Discussion

This study explored depression, anxiety, and stress symptoms in a convenience sample of Ukrainian displaced adults approximately 2 years after the 2022 full-scale invasion. DASS-21 scores in this sample fell within the mild to moderate range and were generally higher than available published European general-population reference values. Within the sample, local language proficiency and employment status were not associated with depression, anxiety, or stress. Housing stability showed a tentative association with distress, with the clearest post-hoc evidence indicating lower anxiety among participants reporting long-term housing compared with medium-term housing. These findings should be interpreted cautiously given the cross-sectional design, convenience sampling, high attrition, variable-specific exclusions due to uncategorizable responses, and the absence of direct measures of trauma exposure, uncertainty, or temporariness.

### 5.1 Elevated psychological distress in Ukrainian displaced persons

Depression, anxiety, and stress scores in the Ukrainian displaced sample were generally higher than available published European general-population reference values from the United Kingdom, Portugal, Poland, and Italy. All benchmark comparisons remained statistically significant after Bonferroni correction except the anxiety comparison with Poland. However, these comparisons should be interpreted as contextual benchmarks rather than matched population comparisons, given differences in sampling frame, year of data collection, country context, gender composition, and measurement conditions across studies. The supplementary sex-composition sensitivity analysis further indicated that the depression comparison was robust when the pooled Ukrainian sample was compared with Polish female reference values, whereas the anxiety comparison was attenuated and the stress comparison was modest. Taken together, these findings suggest elevated distress in this convenience sample, particularly for depression, while underscoring the need for caution in interpreting benchmark differences.

The psychological distress observed in this sample is consistent with literature documenting elevated mental health burden among conflict-affected and displaced populations (8), and with early reports of psychological distress among Ukrainian displaced persons during the crisis phase (1, 23). Mean DASS-21 scores in the present sample fell within the mild to moderate range according to established interpretive guidelines. This indicates clinically relevant symptom burden at the group level, but these scores should not be interpreted as diagnostic prevalence estimates.

Exploratory analyses also examined whether settlement-related factors were associated with psychological distress. Housing stability showed a tentative association with psychological distress. Omnibus tests indicated differences in depression and anxiety across housing-stability categories, although the clearest post-hoc evidence was for anxiety: participants reporting long-term housing had lower anxiety scores than those reporting medium-term housing. Post-hoc comparisons for depression did not remain statistically significant after Holm correction, and differences in stress scores did not reach conventional levels of statistical significance. Given the cross-sectional design, and uncategorizable responses in the housing variable, these findings should be treated as hypothesis-generating.

This pattern can nevertheless be viewed in relation to structural characteristics of housing arrangements for displaced persons, while recognizing that the present data cannot establish directionality. Evaluations of the Homes for Ukraine scheme highlight that medium-term accommodation, particularly host-based arrangements, is often characterized by uncertainty, dependence, and limited autonomy (41). Displaced persons in such settings may be positioned as “dependent guests,” relying on hosts for access to services and everyday support, which may create hierarchical dynamics and psychological strain.

These conditions are consistent with broader research demonstrating that lack of control, instability, and housing precarity are associated with poorer mental health outcomes among displaced populations (3, 14, 42). Long-term housing, by contrast, may provide greater stability, autonomy, and predictability, which may help explain the lower anxiety scores observed among participants reporting long-term housing. More secure housing may also be associated with lower exposure to ongoing stressors related to uncertainty and dependence, which have been linked to poorer psychological outcomes in displaced populations (11). However, reverse causation is also possible: participants experiencing higher anxiety or depression may have found it more difficult to secure or maintain longer-term housing.

Although language acquisition and employment are widely recognized as important integration outcomes and predictors of social and economic participation (14, 43), the absence of an association with psychological distress in this sample suggests that other factors may have been more closely related to mental health in the context of recent conflict-related displacement. One possible explanation is that the psychological impact of war trauma and forced displacement may overshadow certain integration indicators, particularly those related to longer-term social and economic participation, despite unusually generous access to resources intended to support integration (44). This interpretation aligns with research indicating that trauma exposure can have persistent effects that transcend environmental circumstances (2), although trauma exposure was not directly measured in the present study.

A further contextual factor may also be relevant. At the time of the survey, most Ukrainian displaced persons in Europe lacked a pathway to permanent residence and were therefore living in a condition described as “permanent temporariness” (45). Unlike permanently resettled refugees, displaced Ukrainians in Europe often remain in temporary protection regimes without long-term certainty regarding their future, placing them in a different position from the resettled refugee populations featured in much of the refugee mental health literature (14). These conditions may contribute to persistent psychological strain even where some aspects of integration appear relatively favorable, but future research should measure uncertainty and temporariness directly.

Finally, ongoing family separation and caregiving responsibilities may also contribute to distress. A large-scale survey of displaced Ukrainians across Central and Eastern Europe found that 78% of respondents reported separation from immediate family members and that 24% of households included individuals with specific needs, such as persons with disabilities, serious medical conditions, or older adults (46). Such ongoing stressors may contribute to elevated psychological distress regardless of improvements in language proficiency or other integration indicators.

### 5.2 Clinical and policy implications

These findings carry implications for mental health service planning and resource allocation. The elevation in psychological distress across all domains suggests that Ukrainian displaced persons in this sample may have ongoing mental health support needs. This may be exacerbated by the lack of a pathway to permanence, with many individuals living under conditions of ongoing uncertainty regarding their future, regardless of their integration outcomes. Models that prioritize support based on integration outcomes may therefore overlook displaced persons experiencing psychological distress while appearing to function adequately.

The absence of an association between local language proficiency and employment status with psychological distress suggests that post-migration integration indicators may not always correspond to mental health outcomes. Although integration supports for Ukrainians have been relatively generous compared with those provided to many other displaced populations (47), these measures alone may be insufficient to address the psychological burden associated with war and forced displacement. Notably, the tentative association between housing stability and anxiety suggests that secure and stable accommodation may be an important context for psychological wellbeing in this population, although the present study cannot establish a causal effect.

The highly educated profile of the sample may also be relevant to interpreting the employment finding. Employment status alone does not capture whether participants were working in roles commensurate with their qualifications, whether professional credentials were recognized, or whether they were experiencing occupational downgrading. In a highly educated displaced sample, underemployment may itself be a source of stress, meaning that being employed should not necessarily be interpreted as evidence of economic security or psychological recovery.

Given uncertainty and the absence of clear pathways to permanence may contribute to sustained distress, future studies should measure these constructs directly. More targeted, trauma-informed approaches that address both war-related psychological distress and the effects of prolonged uncertainty may therefore be required. This has implications for healthcare systems in host countries, which may need to consider accessible, low-barrier routes to mental health assessment and support rather than relying solely on help-seeking behavior.

The persistence of elevated distress approximately 2 years post-displacement indicates that mental health support needs are likely to be sustained rather than time limited. Healthcare planners should anticipate ongoing demand for psychological services among Ukrainian displaced populations. They should also ensure access to appropriate trauma-informed support for those who report clinically significant or persistent symptoms. At a policy level, clearer pathways to permanence may help address one source of uncertainty for displaced Ukrainians, although future research should directly test whether changes in legal certainty are associated with subsequent changes in psychological distress.

### 5.3 Limitations

Several limitations should be considered when interpreting these findings. First, the cross-sectional design prevents causal inferences about the relationship between displacement, settlement conditions, and mental health outcomes. Longitudinal research would better illuminate the trajectory of psychological distress and would clarify the relationship between settlement factors and mental health.

Second, a key limitation concerns the non-probabilistic nature of the sampling strategy. The study relied on a convenience sample, a common and often unavoidable approach in research with forcibly displaced populations, particularly in conflict and post-conflict contexts (1, 15). However, because the sample is non-random, the findings cannot be considered representative of the broader displaced population, and all estimates should be interpreted as provisional. The lack of probability-based sampling also limits the validity of statistical inference, meaning that comparisons and significance testing should be treated with caution. Selection bias may have been introduced, as individuals experiencing mental health difficulties may have been either more or less likely to participate in research. Additionally, missing data on country of residence further constrains the ability to examine contextual or location-specific effects. These effects might influence the relationship between settlement conditions and mental health.

Relatedly, the high attrition rate and variable-specific exclusion patterns due to uncategorizable responses limit the generalisability of the findings. Of the 529 participants who began the survey, 280 did not complete the DASS-21, and a further eight were excluded following response-quality screening. Missingness was also concentrated in some settlement variables. Employment status and country of residence were missing primarily among DASS-21 non-completers, and housing stability responses were uncategorizable resulting in exclusion for a substantial proportion of participants. These patterns suggest that the analytic sample may over-represent participants who were able and willing to complete a longer survey and may under-represent those experiencing greater instability, distress, survey fatigue, or structural barriers to participation. The housing analysis should therefore be interpreted as exploratory and potentially affected by selection, rather than as providing a definitive estimate of the association between housing stability and psychological distress.

Third, the comparative component of the study is subject to important limitations. Some of the comparison countries included are not major host countries for the displaced population under study, which may reduce the interpretability of cross-national contrasts. However, this reflects the limited availability of suitable reference data using the same instrument, as comparable DASS-21 population norms are not consistently available for all relevant host countries. As a result, European general population samples were used as the closest available benchmark to contextualize the findings. In addition, several of the comparison measures were collected in earlier years, introducing temporal mismatches that may confound differences observed between groups. Given the scarcity of recent and methodologically comparable datasets, the inclusion of these studies was necessary to enable any form of contextual comparison. These constraints should be considered, and the comparisons should be interpreted as indicative rather than definitive, limiting the strength of conclusions that can be drawn.

Fourth, our choice of settlement indicators may not have captured the complexity of post-displacement experiences. Although the indicators were based on established factors in refugee research (40), other variables such as social support networks, quality of employment, experiences of discrimination, or ongoing ties to Ukraine may play a substantial role in shaping mental health outcomes but were not assessed nor included in the present analysis. The sample also included a small number of participants who were living in Ukraine at the time of the survey and reported forced displacement (*n* = 6). These participants were retained because they met the broader eligibility criterion of having been forcibly displaced from Ukraine. However, their displacement trajectories cannot be reconstructed from the available data; for example, they may have been internally displaced throughout, returned to Ukraine after time abroad, or experienced multiple moves. Their current context may therefore differ from that of participants living in host countries abroad, particularly in relation to host-country language proficiency, legal status, and exposure to ongoing conflict. The subgroup was too small to analyze separately.

We also acknowledge the predominantly female composition of our sample (80.5%). While this represents a limitation in terms of gender balance, it also reflects the demographic reality of displacement from Ukraine following the 2022 invasion. Due to martial law and mobilization policies, men aged 18 to 60 have generally been required to remain in Ukraine, meaning that women and children constitute the majority of those displaced abroad. UNHCR data indicate that women and children account for approximately three quarters of refugees from Ukraine, reflecting the demographic structure of displacement under these conditions (48). In this respect, the gender distribution of the present sample broadly reflects the composition of Ukrainian displaced populations reported in assessments. At the same time, family separation and concern for relatives remaining in conflict zones may represent an additional source of psychological strain for many displaced women.

Fifth, the study did not directly measure trauma exposure, bereavement, family separation, witnessing violence, or other war-related experiences. As a result, we cannot determine the extent to which variation in depression, anxiety, or stress reflected prior trauma exposure, current settlement conditions, or their interaction. Similarly, although uncertainty and “permanent temporariness” provide useful contextual frameworks for interpreting the findings, these constructs were not directly operationalized in the survey. Future studies should measure legal uncertainty, perceived temporariness, trauma exposure, and ongoing transnational stressors directly.

Finally, although the comparison to general population norms provides useful contextualization, they should be interpreted cautiously. Such comparisons do not account for potential cross-cultural differences in DASS-21 response, nor for possible differences between pre-war Ukrainian norms and those of the European comparison countries. Additionally, variation in the timing of data collection across reference studies introduces further uncertainty into these comparisons.

### 5.4 Future research directions

Future research should prioritize longitudinal designs to track the trajectory of mental health outcomes among Ukrainian displaced persons over time. Such designs could identify critical periods for intervention during which psychological distress is most acute. Longitudinal work would also clarify whether the observed association between housing stability and mental health reflects a causal relationship, and whether the absence of an association with language competency and employment status persists over time or changes as displacement becomes more prolonged.

In addition, future studies would benefit from employing probability-based or mixed sampling strategies where feasible, to enhance representativeness and strengthen the validity of statistical inference. Where this is not possible, methodological research examining the extent and direction of selection bias in forcibly displaced populations would be valuable.

Further research should expand the range of post-displacement variables assessed to better capture the complexity of settlement experiences. While housing stability emerged as a significant correlate of psychological distress, other structural and psychosocial factors such as social support networks, perceived discrimination, employment quality, and transnational ties to Ukraine may also play a critical role. Examining these factors alongside housing could help clarify the relative importance of material versus social determinants of mental health.

Research examining protective factors specific to this population would be valuable. This includes access to pathways of permanence, the role of cultural factors, social connections within Ukrainian refugee communities, and types of social support that might buffer against psychological distress. Additionally, intervention research testing trauma-informed approaches tailored to the Ukrainian displacement experience could inform evidence-based treatment recommendations. Given the ongoing nature of the conflict and the prevalence of family separation, interventions may need to explicitly address chronic uncertainty and transnational stressors rather than focusing solely on past trauma.

Comparative research would provide further insight into the role of displacement context. Studies comparing individuals who remain in European host countries with those who return to Ukraine, as well as comparisons with internally displaced persons within Ukraine, could help disentangle the psychological impact of different displacement trajectories and living conditions.

### 5.5 Conclusion

This study suggests that Ukrainian displaced persons continue to experience elevated levels of depression, anxiety, and stress approximately 2 years after displacement. This occurs even among individuals who appear relatively integrated on some indicators within this sample. However, given the cross-sectional design and non-probabilistic sampling approach, these findings should be interpreted with caution and cannot be generalized to the wider displaced population. While housing stability showed modest associations with psychological distress, other indicators such as local language proficiency and employment were not associated with depression, anxiety, or stress symptoms. This pattern provides only partial support for frameworks emphasizing post-migration conditions and highlights the need for further research using more comprehensive measures and representative designs. The findings suggest that approaches focusing solely on integration indicators may be insufficient to capture the factors associated with psychological distress in this population.

Overall, the study highlights the likelihood of sustained mental health needs among Ukrainian displaced populations following forced migration due to armed conflict. While further research is required to establish causal pathways and improve generalisability, the findings support the continued prioritization of accessible, trauma-informed mental health support, alongside efforts to improve settlement conditions, particularly housing stability, within public health responses to displacement.

## Data Availability

The datasets presented in this study can be found in online repositories. The names of the repository/repositories and accession number(s) can be found at: https://osf.io/2qr95/.
