# Supplementary material for: Psychological distress among Ukrainian displaced persons 2 years after the full-scale invasion: a contextual benchmark comparison
Source: Front Public Health. 2026 Aug 19;14:1858781. doi: 10.3389/fpubh.2026.1858781 (PMC13533821; doi:10.3389/fpubh.2026.1858781)
Supplement: Supplementary file 1 [file Supplementary_file_1.docx]

Supplementary Material S1

# Completer versus non-completer analyses

To assess potential response bias, demographic and settlement characteristics were compared between participants who completed the DASS-21 and those who did not. DASS-21 completion was defined prior to additional response-quality exclusions. Comparisons were therefore conducted between DASS-21 completers before exclusions (n = 249) and non-completers (n = 280). A further eight DASS-21 completers were excluded following response-quality screening, resulting in the final analytic sample reported in the main manuscript (N = 241).

Because background and settlement items had variable-specific missingness among non-completers, the number of participants included in each comparison differed across variables. These analyses were intended as descriptive checks on possible response bias and should not be interpreted as ruling out selection effects. A summary of all completer versus non-completer analyses is provided in Supplementary Table S1.

# 1 Supplementary Figures and Tables

## 1.1 Age

Age data were available for all DASS-21 completers before exclusions (n = 249) and for 85 non-completers. Participants who completed the DASS-21 had a mean age of 39.38 years (SD = 10.46), whereas non-completers with available age data had a mean age of 39.54 years (SD = 10.24). A Welch two-sample t-test indicated no significant difference in age between completers and non-completers, t(148.16) = -0.13, p = .899, 95% CI [-2.72, 2.39]. The effect size was negligible, Cohen’s d = -0.02. This analysis is summarised in Supplementary Table S1.

## 1.2 Host-country language competency

Self-rated host-country language competency data were available for all DASS-21 completers before exclusions (n = 249) and for 45 non-completers. Mean self-rated language competency was similar between completers (M = 5.05, SD = 2.52) and non-completers (M = 5.00, SD = 2.67). A Welch t-test indicated no significant difference between groups, t(59.06) = 0.11, p = .911, 95% CI [-0.81, 0.91]. The effect size was negligible, Cohen’s d = 0.02. This analysis is summarised in Supplementary Table S1.

## 1.3 Education

Educational attainment was not significantly associated with DASS-21 completion. Because several education categories had small cell counts, Fisher’s exact test was used rather than a chi-square test. The test indicated no statistically significant association between education level and DASS-21 completion, p = .56. Completion rates were broadly similar across education categories, ranging from 68.1% among participants with a bachelor’s degree to 85.7% among participants with a high school education. This analysis is summarised in Supplementary Table S1.

## 1.4 Employment status

Employment status data were unavailable for 251 participants, all of whom were DASS-21 non-completers. Analyses of employment status were therefore restricted to participants with non-missing grouped employment information. Among those with grouped employment data, DASS-21 completion rates were generally high across most categories: full-time, 93.6%; part-time, 88.9%; self-employed, 93.3%; unemployed or job-seeking, 86.1%; retired, 100%; disabled, 40.0%; and other, 88.2%.

Because several employment categories had small cell counts, Fisher’s exact test was used. Using the seven-category grouped employment variable, there was weak evidence of an association between employment group and DASS-21 completion, p = .047. However, this result was sensitive to sparse categories, particularly the very small disabled and retired groups. When low-frequency categories were collapsed, the association was no longer evident. The employment completion analysis should therefore be interpreted cautiously and primarily as indicating substantial missingness among non-completers rather than a robust employment-related completion effect. This analysis is summarised in Supplementary Table S1.

## 1.5 Housing stability

Housing stability was assessed using a free-text item asking participants how long they could remain in their current housing. A substantial number of participants did not provide a response or provided a response that could not be categorised. In total, 315 participants had missing or uncategorisable housing-stability data; 246 of these participants were DASS-21 non-completers, indicating that missing or uncategorisable housing information was concentrated among participants who did not complete the DASS-21.

Among participants with categorisable housing-stability data before response-quality exclusions, DASS-21 completion rates were 70.0% for short-term housing, 83.6% for medium-term housing, 86.4% for long-term housing, and 87.0% for uncertain housing. Fisher’s exact test indicated no statistically significant association between housing-stability category and DASS-21 completion, p = .31. This analysis is summarised in Supplementary Table S1.

The substantive housing-stability analyses reported in the main manuscript were conducted only among participants in the final analytic sample who had categorisable housing-stability responses. This resulted in an analytic housing-stability sample of N = 174. Housing-stability descriptives are reported in Table 3 of the main manuscript.

## 1.6 Gender

Gender data were available for 334 participants. Of participants identifying as women (n = 262), 75.6% completed the DASS-21; among participants identifying as men (n = 68), 69.1% completed the DASS-21. The one participant identifying as non-binary and the three participants who preferred not to state their gender completed the DASS-21. Because of small cell counts in several categories, Fisher’s exact test was used. The test indicated no statistically significant association between gender and DASS-21 completion, p = .55. This analysis is summarised in Supplementary Table S1.

## 1.7 Country of residence

Country of residence data were available for 306 participants. Country data were missing for 223 participants, all of whom were DASS-21 non-completers. This indicates that missing country information was strongly concentrated among participants who did not complete the DASS-21.

Among participants who provided country information, countries with larger numbers of respondents were retained as separate categories, while countries with small numbers of respondents were collapsed into an “Other small-country groups” category. Country group was not significantly associated with DASS-21 completion, χ²(8) = 4.89, p = .769. Completion rates were broadly similar across the larger country groups, suggesting no clear evidence of country-specific differences in DASS-21 completion among participants who provided country information. This analysis is summarised in Supplementary Table S1, with full country-group completion data reported in Supplementary Table S2.

# Supplementary Table S1

Summary of completer versus non-completer analyses

| **Variable** | **Available comparison** | **Test** | **Result** |
| --- | --- | --- | --- |
| Age | Completers n = 249; non-completers n = 85 | Welch t-test | t(148.16) = -0.13, p = .899 |
| Host-country language competency | Completers n = 249; non-completers n = 45 | Welch t-test | t(59.06) = 0.11, p = .911 |
| Education | Participants with available education data | Fisher’s exact test | p = .56 |
| Employment status | Participants with grouped employment data | Fisher’s exact test | p = .047; sensitive to sparse cells |
| Housing stability | Participants with categorisable housing-stability data | Fisher’s exact test | p = .31 |
| Gender | Participants with available gender data | Fisher’s exact test | p = .55 |
| Country group | Participants with country data | Chi-square test | χ²(8) = 4.89, p = .769 |

# Supplementary Table S2

DASS-21 completion by country group among participants with country data

| **Country group** | **Non-completers n** | **DASS-21 completers n** | **Total n** | **Completion %** |
| --- | --- | --- | --- | --- |
| United Kingdom | 15 | 75 | 90 | 83.3 |
| Germany | 6 | 37 | 43 | 86.0 |
| Spain | 9 | 26 | 35 | 74.3 |
| Poland | 8 | 27 | 35 | 77.1 |
| Portugal | 3 | 15 | 18 | 83.3 |
| Croatia | 1 | 9 | 10 | 90.0 |
| Slovenia | 2 | 8 | 10 | 80.0 |
| Ukraine | 3 | 5 | 8 | 62.5 |
| Other | 10 | 47 | 57 | 82.5 |
| Total | 57 | 249 | 306 | 81.4 |

Note. Country data were missing for 223 participants, all of whom were DASS-21 non-completers. Participants from England, Scotland, Wales, and Northern Ireland were grouped as United Kingdom. Countries with small numbers of respondents were grouped as “Other”.
