# Supplementary material for: Psychological distress among Ukrainian displaced persons 2 years after the full-scale invasion: a contextual benchmark comparison
Source: Front Public Health. 2026 Aug 19;14:1858781. doi: 10.3389/fpubh.2026.1858781 (PMC13533821; doi:10.3389/fpubh.2026.1858781)
Supplement: Supplementary file 2 [file Supplementary_file_2.docx]

Supplementary Material S2

# Supplementary Material S2

# 1 Sex-composition sensitivity analysis for benchmark comparisons

The Ukrainian displaced sample was predominantly female. This raises the possibility that some differences between the Ukrainian sample and published benchmark samples could partly reflect differences in sex composition rather than differences associated with displacement context. To examine this possibility, we reviewed the published benchmark studies used in the main manuscript to determine whether sex-specific DASS means and standard deviations were available.

Sex-specific DASS means and standard deviations were available only for the Polish reference sample (39). The United Kingdom, Portuguese, and Italian reference studies did not report sex-specific DASS subscale means and standard deviations sufficient for equivalent analyses. We therefore conducted a supplementary sex-composition sensitivity analysis using the Polish female reference values.

We did not stratify the Ukrainian sample by sex because the sample was predominantly female and the male subgroup was comparatively small, making sex-stratified estimates within the Ukrainian sample unstable. Instead, we compared the pooled Ukrainian sample with the Polish female reference values. This provides a conservative sensitivity comparison because the pooled Ukrainian mean includes male participants, whereas the Polish female reference means are higher than the Polish mixed-sex reference means.

The sensitivity analysis used the same summary-statistic Welch t-test approach described in the main manuscript. The Ukrainian DASS-21 subscale scores were multiplied by two to align with the full DASS scoring metric used in the benchmark comparisons.

As shown in Supplementary Table S3, the depression comparison remained clear when the pooled Ukrainian sample was compared with the Polish female reference values. The anxiety comparison was attenuated and was not statistically significant. The stress comparison was modest and should be interpreted cautiously. Because this analysis was conducted as a supplementary sensitivity check rather than as an additional primary benchmark family, results are interpreted descriptively. If a Bonferroni correction were applied across all 15 benchmark and sensitivity comparisons reported across the main manuscript and Supplementary Material S2, α would be .00333; under this stricter threshold, only the depression comparison with the Polish female reference values would remain statistically significant.

## 1.1 Supplementary Table S3

## Sex-composition sensitivity analysis comparing the pooled Ukrainian sample with Polish female reference values

| **DASS subscale** | **Ukrainian pooled sample M (SD), N = 241** | **Polish female reference M (SD)** | **Mean difference** | **Welch t(df)** | **p** |
| --- | --- | --- | --- | --- | --- |
| Depression | 15.55 (10.52) | 9.86 (8.52) | 5.69 | t(367.64) = 7.50 | < .001 |
| Anxiety | 10.59 (9.56) | 9.48 (7.70) | 1.11 | t(366.41) = 1.61 | .108 |
| Stress | 17.93 (10.85) | 15.93 (8.98) | 2.00 | t(373.52) = 2.54 | .012 |

Note. Ukrainian DASS-21 subscale scores were multiplied by two to align with the full-scale DASS scoring metric used in the published benchmark comparisons. Polish female reference values were used because sex-specific means and standard deviations were available only for the Polish benchmark study. This analysis was conducted as a descriptive sensitivity check for sex composition and should not be interpreted as a matched population comparison.
